# An Innovative Method for Preserving Privacy in Internet of Things

**DOI:** 10.3390/s19153355

**Published:** 2019-07-31

**Authors:** Mohammad Yamin, Yazed Alsaawy, Ahmad B. Alkhodre, Adnan Ahmed Abi Sen

**Affiliations:** 1 Department of Management Information Systems, Faculty of Economics and Administration, King Abdulaziz University, Jeddah 21589, Saudi Arabia; 2 2 Faculty of Computer and Information Systems, Islamic University, Al-Madinah 42351, Saudi Arabia

**Keywords:** privacy, blind approach, Internet of Things, Blind Third Party, Blind Peers, Smart City, E-health, encryption, sensors, underwater sensor networks, transportation, Smart Homes

## Abstract

Preservation of privacy of users’ personal data has always been a critical issue to deal with. This issue in the Internet of Things (IoT), which facilitates millions of applications, has become even more challenging. Currently, several approaches and methods are available to safeguard privacy but each of them suffers from one or more anomalies. In particular, Trusted Third-Party approach relies on the trust of a third-party server, Cooperation needs the trust of other peers, Obfuscation is known to return inaccurate results, and Dummy generates too much overhead. Moreover, these and most of the other well-known approaches deal only with specific types of applications linked to the location-based services. In this paper, we present two new methods, namely: Blind Third Party (BTP) and Blind Peers ($B_{L}P$), and combine them to form a new one to be known as the Blind Approach ($B_{L}A$). With the help of simulation results we shall demonstrate the effectiveness and superiority of $B_{L}A$ over the other available methods. The simulation results also exhibit that $B_{L}A$ is free from all the existing problems of the other approaches. However, $B_{L}A$ causes a slight increase in the average (response) time, which we consider to be a minor issue. We shall also discuss the capability and superiority of the Blind Approach in the cases of E-health, Smart Transportation, and Smart Home systems.

## 1. Introduction

**Figure 1:**
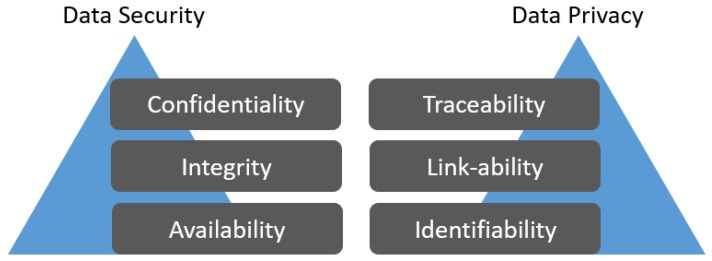
Privacy vs. Security.

Privacy is about the right of people to determine how, where, when, and who can use their data. Preserving privacy is about preventing misuse of personal data, which normally includes identity and the associated information about location, health, social status, financial condition, contact details, etc. Personal information is often sensitive [1] and unsolicited use of it may result in serious and sometimes life-threatening consequences. In Internet of Things (IoT) applications, users provide personal data to different devices and services, where privacy becomes extremely vulnerable [2,3,4]. In particular, location-based service (LBS), which uses personal data of users, is becoming increasingly popular [5]. Preservation of users’ identity (and hence the personal information) from the service providers (SPs) has attracted the attention of many researchers. Privacy and security are different concepts which we have compared and depicted in Figure 1.

### 1.1. Background

**Figure 2:**
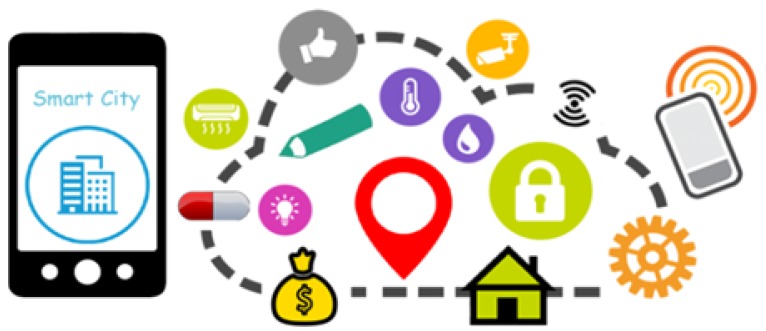
IoT Application.

The IoT [6] is now contributing to solving many issues and problems arising in business, industry, science and socio-economic areas to positively and significantly improve the quality of our lives [7,8]. IoT is a network of many devices dominated by Wireless Sensor Networks (WSNs), Radio Frequency Identification Tags (RFID Tags), and Smart Devices [9,10,11]. These devices can sense and identify everything in one’s body or the surrounding environment to provide the required data for several innovative services and applications such as smart cities, smart home, E-health systems, E-learning, E-business, Location-based Services (LBS), crowd management, etc. as depicted in Figure 2. The objects (things) of IoT are growing at a very fast rate and are expected to grow to several billions by 2020 [11,12,13]. Therefore, we can expect an even larger number of applications and more benefits from these technologies. The objects of IoT sense massive data of users and send it to the SP, which is stored in clouds, resulting in significant privacy issues, which must be addressed properly and adequately [14]. As the number of IoT devices increases, so does the vulnerability to privacy breaches. Effective and concerted measures to ensure privacy is paramount to boost user confidence in dealing with their data in IoT applications or systems [15,16]. Preservation of privacy of user identity and associated data is about protecting it not only from outsider intruders but also from insider breaches, dominated by SPs, involving profiling, tracing, collecting, or unauthorized usage [17,18,19].

Many researchers are engaged in elucidating and exploring different ways and methods to ensure privacy. The available methods and approaches for preserving privacy of data in IoT applications, without any exception, suffer from one or more shortcomings, and hence the problem of ensuring the privacy of data is far from being resolved. A survey of available methods to ensure privacy in IoT applications [20] reveals that many of them are limited to LBS-enabled applications [21,22,23]. Moreover, these approaches are reliant on the third trusted party (TTP), which can’t be taken for granted [24]. The SP or TTP can indeed be the internal attackers or partner to a host of external attackers [25]. Furthermore, the users are forced to compromise on the accuracy of their retrieved results [26]. A few methods have also dealt with the privacy of E-Health data, mostly involving either discrete queries or security rather than privacy [27]. Invariably, these methods force the users to absolutely trust the SP, which can be regarded as having next to no privacy protection.

**Figure 3:**
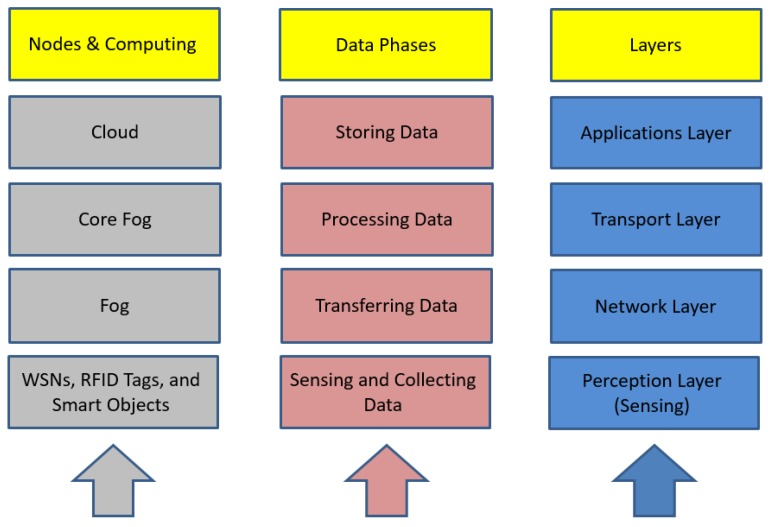
IoT layers and phases.

Data in IoT applications passes through four (main) phases, each with several layers [28,29], as depicted in Figure 3. These four phases are Sensing and Collecting, Transferring, Processing, and Storing. Any protection scheme or method must ensure privacy in all these phases. The figure also shows three types of computing models, namely: cloud, fog, and core fog. It is worth mentioning that fog nodes are densely distributed at the edge of the network. Fog is used to enhance the speed of the response and capability of IoT applications, where the central cloud may be sluggish in response because of bottlenecks. Moreover, the usage of fog can reduce the dependence on trust with the SP [2]. Core fog is no different to fog except that it has more resources, and sits in between cloud and fog for better management.

**Figure 4:**
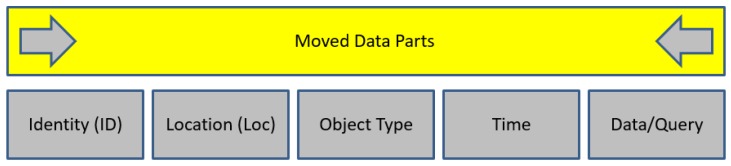
Main parts of generic IoT query.

The mobile data during and after the transition, as shown in Figure 4, has five parts, namely: Identity, Location, Type, Time, and Data/Query, the most sensitive of which is determined by the nature of the ensuing application. To propose a protection scheme or technique, it would be logical to focus on the sensitive parts rather than all of them to promote a considerable trade-off between privacy and performance [30,31].

Ensuring the security of application data from outsider attacks can be dealt effectively through cooperation between the user and the trusted SP by encryption [32,33,34]. However, protection of privacy of the user deals with both the outsider intruders and insider attackers, one of which could be the SP itself. To safeguard the privacy of personal data from the SP is a vexed issue because most of the available methods resort to adding noise to data, which adversely impacts accuracy of the results, rendering it to be unacceptable in some domains like health.

### 1.2. Statement of Problems and Our Contribution

The problem of preserving privacy has become critical in IoT applications involving personal data of users. Existing methods for the protection of privacy are not reliable for one reason or another. The most prominent of the existing methods, namely: Trusted Third Party, Cooperation, Obfuscation, Dummy, Data Encryption, Private Information Retrieval (PIR) and Caching approaches, to be referred as the ’group of seven’ approaches, suffer from various kinds of anomalies. For example, Trusted Third Party is reliant on a third-party server, Cooperation is built on the trust of other peers, Obfuscation is prone to produce inaccurate results, and Dummy has a problem of generating strong dummies in addition to resulting in too much overhead such as that in PIR. Also, as mentioned earlier, the group of seven approaches deal only with the LBS. Thus, there is no known method which can be relied upon for the protection of privacy in IoT.

In this article, we propose a new method to be known as the Blind Approach, abbreviated as $B_{L}A$, to protect the user’s identity and associated personal data in the IoT applications. The Blind Approach works by way of effectively misleading SP about the real identity and query of the user, and unlike the group of seven approaches, neither relies on the trust of any party nor compromises the accuracy of the results, with only a small overhead. The $B_{L}A$ is built by combining two methods, namely: Blind Third Party (BTP) and Blind Peer ($B_{L}P$), and also presents an adaptive scenario between BTP and $B_{L}P$, to be referred to as Integrated Blind Parties (IBPs). Blind Approach offers a solution to all the existing problems found in the in the previously mentioned group of seven approaches. In this article, we have achieved the following:

1. A detailed description of how $B_{L}A$ preserves the privacy in IoT applications
2. A discussion of the relevance of BTP, $B_{L}S$, and IBPs, and usefulness and effectiveness of
  (a) BTP in overcoming the drawbacks in Trusted Third-Party (TTP) approach
  (b) $B_{L}P$ in addressing the problem of trust among peers in the Cooperation approach
  (c) IBPs to creating dynamism between BTP, $B_{L}S$
3. A detailed analysis of attacks to demonstrate the advantages of in Blind Approach in safeguarding privacy without affecting the accuracy of results or data
4. Applications of $B_{L}A$ for preserving privacy of users in E-health, Transportation, and Smart Home systems, as well as underwater sensor networks, without having to trust any third party.

## 2. Literature Review

**Figure 5:**
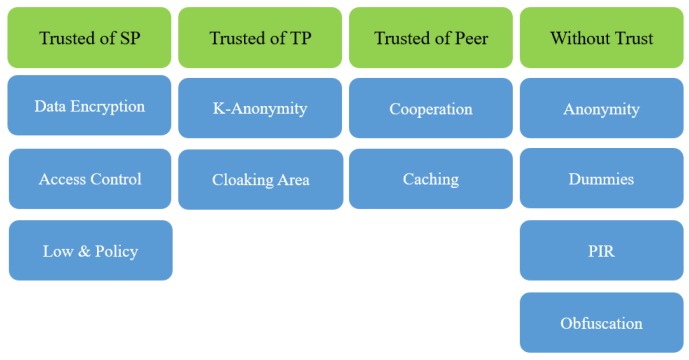
Classification of IoT privacy approaches.

In this section, we shall discuss all significant approaches for preserving privacy in IoT. We have grouped them in four categories based on the trust issue [22] as shown in the Figure 5. An additional group-based partially on trust or on semi-trust can also be added into this classification, whereas the fourth group, namely: ’Without Trust’, can also be classified as ’Trust Free’. Researchers over the years have invented several approaches to and methods for each group. Here we shall outline main ideas together with the drawbacks in each of them. In keeping up with the scope of this article, we shall focus on the group of seven approaches, members of which suffer from cooperation between peers, trust in the third party (TP), too much overhead, or adverse effects on the result provided by the service.

As shown in Figure 5, we have selected 11 approaches, grouped in four categories. Here we provide a brief description of each of them.

### 2.1. Date Encryption

This method sends user data to SP as encrypted strings. Other methods dealing with data apply Data Mining or Statistical functions. In this approach, processing and filtering of data is undertaken before sending it to a service provider (SP). Data mining and statistical models are examples of data analytics methods used in this approach. A drawback of this approach is that it can be used only in applications where the sensitive data is not involved [35]. The cases where encryption is used for protection of user’s data from attacker are dealt with the trusted SP.

### 2.2. Access Control

When using this approach, the user would receive a notification whenever SP wanted to use their data. Although the users would have full access and control of their data but there is no surety that SP wouldn’t violate the privacy of the user [22,36,37,38].

### 2.3. Law and Policy

It is a measure which requires the user to be aware of their privacy as well as the policy of SP [38,39].

### 2.4. Clocking Area and K-Anonymity

In these methods, a broker or mediator server, between users and SP is used to protect users’ privacy in a specific area from SP by hiding their identities (Anonymizer), creating a homogeneous cluster for users, storing some data in cache for answering future queries without connecting to SP, or storing historical information of users to predicate their transaction and then obfuscate it. Details of this method can be found in [20,22,24,40]. Although the users wouldn’t be required to trust SP, they instead would have to trust TP.

### 2.5. Cooperation

User is required to cooperate with another peer or several peers to protect privacy. As a result, peers are required to swap their queries and answers, storing some answers in cache, transmitting the same query, or creating shared clacking area. How the peers can be trusted is still an open problem [41].

### 2.6. Caching

User must rely on the cache, where many of the previous highly requested queries’ answers are stored to offset the number of connections with the SP. This approach can be integrated with other approaches to enhance the privacy level. However, determination of a suitable position for cache and the issue of trust are still wide-open problems [42].

### 2.7. Anonymity

A user sends the query with nickname or using hashing code instead of his identity. But it is not effective and easy to be hacked by attacker. To enhance this approach, users are required to change their nickname each time or whenever they enter a new area to prevent their tracing by SP. This is known as Mix-zone, which isn’t very effective to protect the privacy of users because SP can easily trace the location and reconnect with a new nickname [43].

### 2.8. Obfuscation

In this case, the users adulterate their data and by adding some noise before sending it to SP to protect themselves. As a result, this method would affect the accuracy of results and the performance wouldn’t be optimum [44]. Other aspects of this approach are related to the TTP approach, as described above.

### 2.9. Dummies

A user sends a group of queries (K) to the SP instead of just one, (K-1) of those will be dummies and the remaining one will be the actual query. That is done to prevent the SP from profiling the user information. The drawback of this approach lies in the fact that generation of effective dummies isn’t a complex task, and the process adversely impacts the performance as well. For details, refer to [45].

### 2.10. Private Information Retrieval (PIR)

User retrieves a big part of data from the SP about a specific area/block, and store it safely for future queries. Moreover, this data can be encrypted or distributed to many servers to increase the level of privacy. This process severely affects the performance and creates an accumulated backlog of data for users to store and handle it [46].

There is also another method called Hybrid which uses more than one technique to protect the privacy. Examples are caching with dummies, encryption with obfuscation, and so on [47,48].

From the preceding discussion, it is evident that the real problem in all of these approaches is that of the trust in another party which can be the SP, TP, or Peer, which are capable of breaching privacy and so the user would remain vulnerable in every situation. Attempts to relieve users have resulted in more problems to them and, in addition, adversely effected the accuracy of results [22,35].

To address these issues completely, our approach, proposed in this article, addresses three scenarios, namely: Blind Third Party (BTP), Blind Peer $B_{L}P$, and Integrated Blind Parties (IBPs). In our approach, we have successfully eliminated need for trust with any of the above-mentioned parties, without impacting on the precision of results. Moreover, this approach can be used in different applications of IoT, not just the location-based services applications.

## 3. Proposed System

Inspiration for the proposed approach came from trying to answer the question: Why do we have to trust the third party to protect the user from the SP? Of course, in order to avoid trusting SP, we shift the problem of trust from one party (SP) to another (TP/Server), which sometimes is easier to deal with. In dealing with TP/Server in $B_{L}A$, we define a new protocol for dealing with SP.

**Figure 6:**
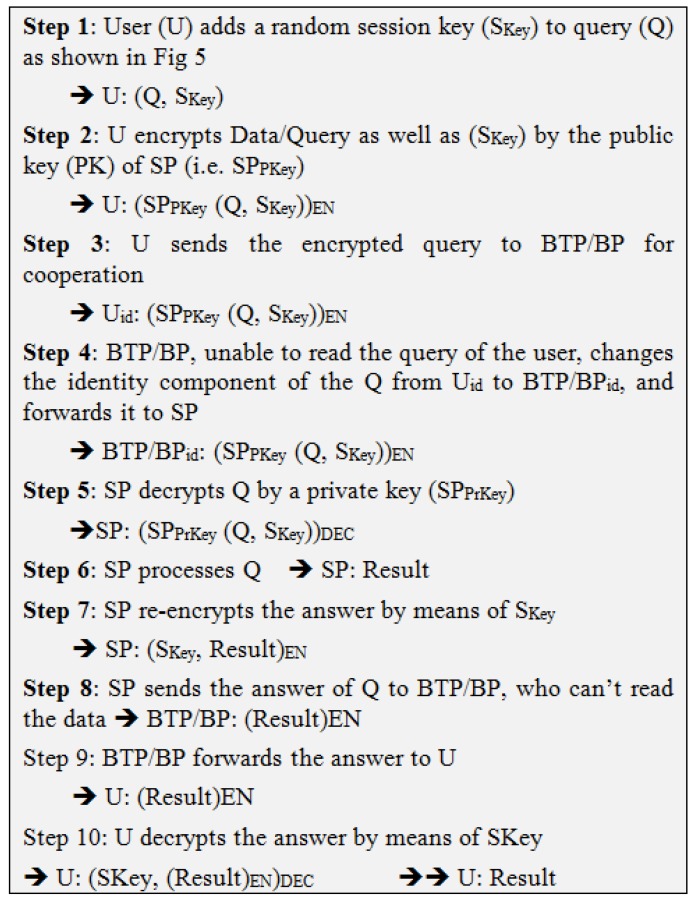
Steps of the query transaction in $B_{L}A$.

**Figure 7:**
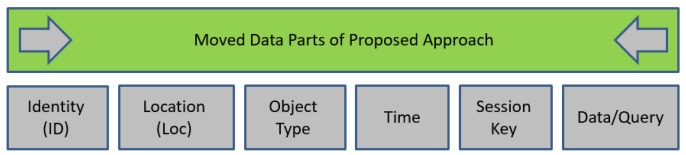
Parts of generic query in Blind Approach.

As for the protection from TP/Server, we propose encryption with two different types of keys, namely: Public/Private Keys and Session Keys to hide the identity of the user from SP and that of the data from the mediator (TP/Server). These measures enable users to reap all the benefits flowing from the usage of TP/Server without creating any avenue for breach of data. Therefore, TP/Server can be regarded as blind and that is why we call it Blind TP/Pear or BTP/$B_{L}P$. Steps of the query transaction in $B_{L}A$ are summarized in Figure 6. Our data model is depicted in Figure 7. It should be noted that we change general format of IoT query by adding to its session key as a new part.

Here we provide three scenarios of the proposed approach in detail.

### 3.1. First Scenario—Blind Third Party (BTP)

This scenario provides a solution to an earlier drawback of exposing data in the TTP approach above (E). In the proposed BTP approach, the user would avail all the benefits of using the TP without having to reveal any data. For that reason, we have renamed TP to BTP. This scenario makes the traditional TTP approach usable by removing its anomaly (trust in TP). The user can use this scenario to expedite the response even in the absence of other peers in the same area or with low resources.

**Figure 8:**
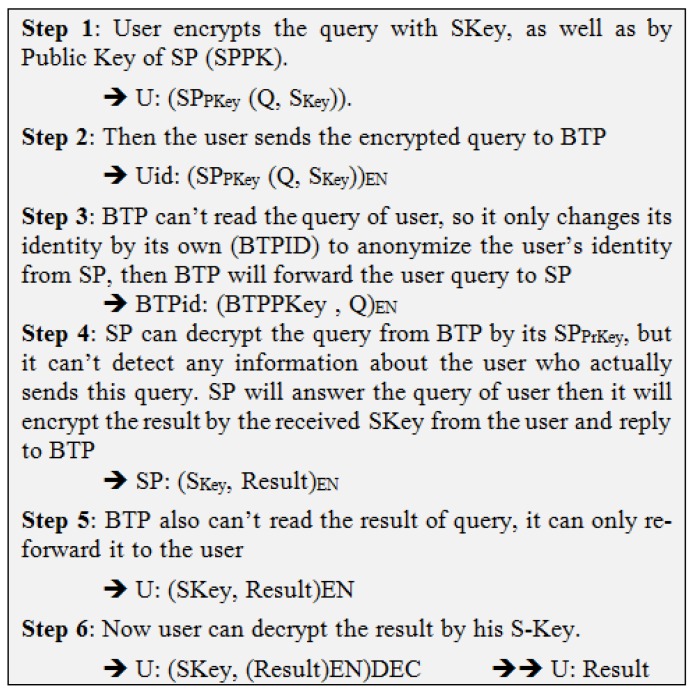
The BTP scenario.

**Figure 9:**
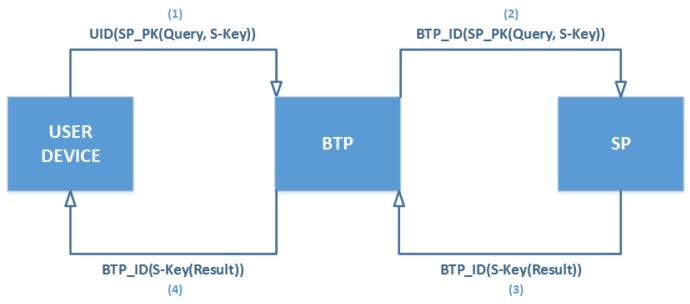
Blind Third-Party Technique (BTP).

This scenario is depicted in Figure 8, and the method is presented in Figure 9.

#### 3.1.1. Advantages of BTP Approach

- The approach provides full protection for user privacy from SP as it can’t track the user because BTP would relay the queries of all users in the area/cell.
- There would be full protection for the data of the user from all outer attackers because the data would be encrypted in both directions.
- BTP itself can’t penetrate into the privacy of users who rely on it.
- The user would connect only with the BTP and hence there wouldn’t be any overload on the user.
- BTP’s role is only to change the identities of users, and hence would be moderately loaded.
- There wouldn’t be additional load on the link, as the number of connections with the SP would stay the same.
- The BTP approach establishes an integration between TTP and Encryption approaches.
- The BTP approach is also capable of supporting IoT applications in which sensors frequently output data which is saved in the SP, and which involve steady stream of queries (not just the discrete ones), and generation of fake IDs which are repeatedly used for the same user availing similar services. An example of such a case is discussed in Section 8.

#### 3.1.2. Disadvantages of BTP Approach

- There is a possibility of collusion between the BTP and SP to breach user’s privacy; however, as the users normally deal with credible service providers, this would be highly improbable. Nevertheless, this disadvantage can be considered to be the main drawback of the BTP approach.
- Encryption may cause overload on some user’s devices; however the proposed approach tries to relax this anomaly by using symmetric keys for encryption of the larger amount of data (i.e., the result of the query) and not relying on asymmetric keys in either direction.
- Using encryption would result in more power consumption by users’ devices

### 3.2. Second Scenario—Blind Peer Approach

**Figure 10:**
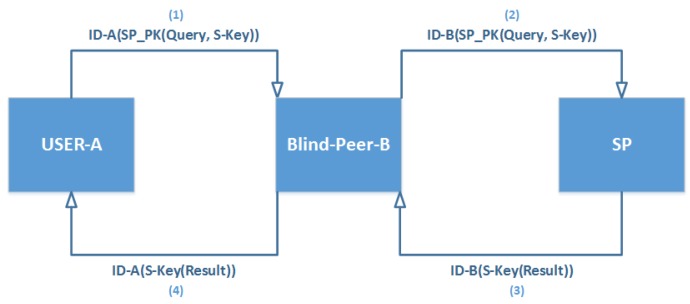
Blind Peer Technique ($B_{L}P$).

**Figure 11:**
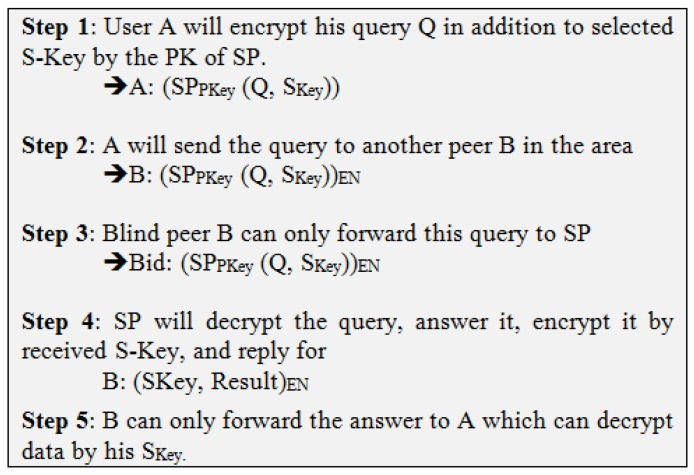
Blind Peer ($B_{L}P$) Approach.

This scenario addresses the main drawback of the previous scenario in which BTP may cooperate with SP to violate the privacy of the users. In the $B_{L}P$ approach, the new idea is to rely on cooperation with many peers instead of dealing with just the same TP. Therefore, a user’s query would be exchanged with another peer in the same area, and subsequently be encrypted by PK of SP ($SP_{PK}$), leaving the other peer with only the option to forward the query to the SP, who would decrypt and resolve the query. The SP would then encrypt the answer by the Session Key ($S_{Key}$) and send it to the peer, while being unable to read the answer, would forward it to the main user. The same scenario would be repeated with peers’ other queries as depicted in Figure 10. The steps in Figure 11 elaborate this method.

While in $B_{L}P$, the user seeks more privacy vs. response time, because in the previous scenario the BTP can deal with the SP to disclose the users’ privacy, even though this would be a rare possibility in real life. But searching for other peers in the hope of resolving the query, can affect the response time. Therefore, this scenario would depend on the cooperation between peers and swapping mechanism (P2PCache) but only after integrating it with Blind Concept and applying $B_{L}A$ protocols, can it avoid its disadvantages.

#### 3.2.1. Advantages of Blind Peers

- All advantages of BTP and P2PCache Approach [47] are retained and all disadvantages are removed. In particular, $B_{L}P$ removes the possibility of collusion between BTP and SP, because each time the user would deal with a different peer.
- There would not be any additional load on any peer and there would be no bottleneck in the TP.

#### 3.2.2. Disadvantages of Blind Peers

- $B_{L}P$ Approach would have same disadvantages as in BTP, except for the possibility of collusion between BTP and SP.
- Occasionally, a suitable peer for cooperation may not be available.

### 3.3. Third Scenario—Integrated Blind Parties (IBPs)—Adaptive Scenario

**Figure 12:**
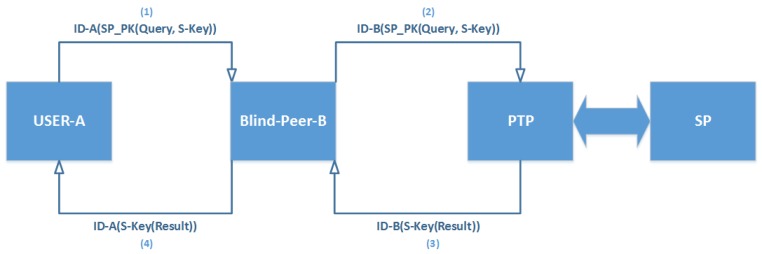
Integrated Blind Parties (IBPs).

This IBPs approach doubles the level of privacy by way of integration between the BTP and $B_{L}P$, and removes the shortcomings of the group of seven approaches. In this approach, the user can rely only on the $B_{L}P$ if a peer isn’t available in the area. Moreover, the user can swap the query with another peer without encryption in case of scarcity of resources. In that case the peer can apply the BTP scenario instead of the user doing it. This scenario can also be adapted in the group of seven approaches [47]. Figure 12 depicts steps of IBPs. We could also use Cache with Bloom filter (to be discussed in Algorithm 2) to enhance the performance in the same way as is proposed in the DCA method [48].

### 3.4. Algorithm of IBPs

**Figure 13:**
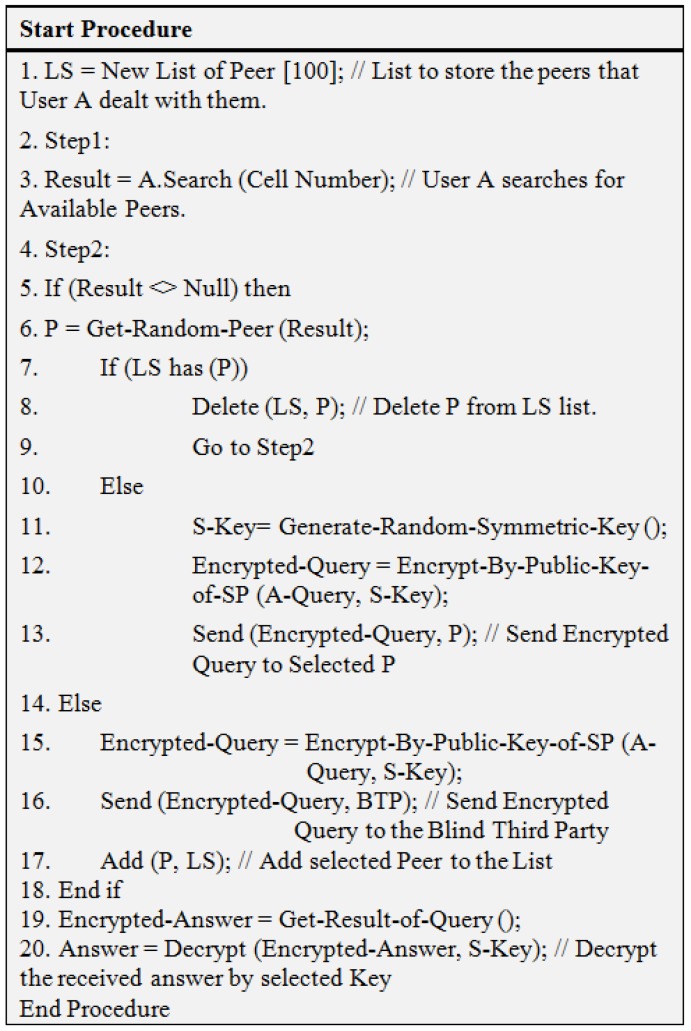
Algorithm 1 of IBPs.

**Figure 14:**
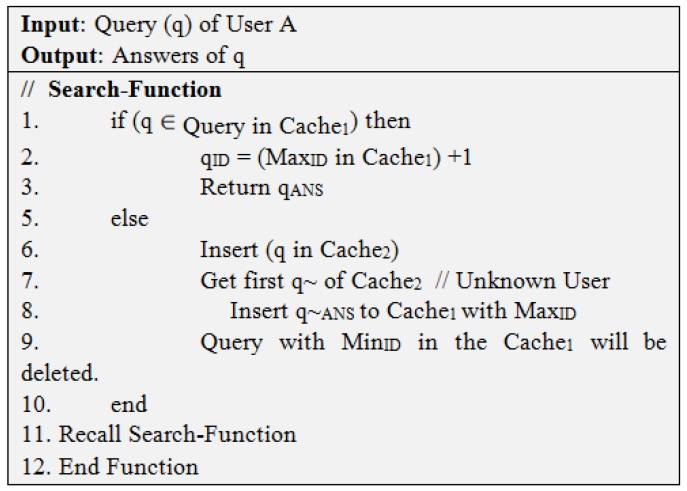
Algorithm 2 of IBPs.

We provide two algorithms of IBPs. First algorithm of IBPs (Algorithm 1) is provided in Figure 13 and second (Algorithm 2) is provided in Figure 14.

## 4. Analysis of Attacks in Blind Approach

Here a discussion on the resilience of the three kinds of the Blind approaches, and the kind of attacks [22,24,27,48] they can deal with, follows.

### 4.1. Resilience of BTP, Blind Peers and IBPs

The proposed techniques in the Blind Approach can deal with many types of attacks/skills of attackers as follows.

#### 4.1.1. Semantic Context Attack

In cases where the attacker has additional information such as user’s age, profession, it may not be too difficult to detect the owner of the query. In BTP and $B_{L}P$, queries are routed by the BTP or $B_{L}P$, therefore there wouldn’t be any connection between the user and SP, and hence the user would be immune to attacks by the SP.

#### 4.1.2. Trajectory Attack

SP can track the user’s positions to trace their path of their movement, then SP would detect new position of the user even if the user adds some obfuscation to it.

#### 4.1.3. Historical (Temporal) Attack

A Historical attack can take place if the attacker has historical information. This cannot happen in cases of BTP and $B_{L}P$ as the SP wouldn’t have the credentials to extract historical information about users.

#### 4.1.4. Inversion Attack

An Inversion Attack can take place if the attacker can extract information about users from the algorithms used. However, in the cases of BTP and $B_{L}P$, the only available information would be the peers routing the queries to the SP, and hence information about the users wouldn’t be detectable.

In addition to the above analysis, $B_{L}P$s offer a superior and new level of resistance to physical attacks on peer devices. To illustrate this, consider that a user exchanges data with a peer p. Suppose that some malicious code manages to erase the memory of P before U gets the answer back. Unable to determine the identity, P would be unable to send the answer back to U. In this case P can simply broadcast the answer to all peers in the cell, enabling U to decrypt the answer, which would be disappointing for the attacker as they would fail to enforce Denial of Service (DoS) in the Blind Approach.

### 4.2. Enhanced Protection in BTP, Blind Peers and IBPs Approaches

The BTP, $B_{L}P$ and IBPs techniques also achieve a great deal of enhancement of the level of protection and are designed to prevent an attacker from breaking in depending on some skills. A discussion on this follows [22,48].

#### 4.2.1. Closeness (Uniformity)

Closeness is a property which is achieved when the areas covered by two different BTPs are similar to each other. This can be realized by having only one BTP in each cell.

#### 4.2.2. Diversity

For Diversity to take place, queries coming from any cell must be different. In the case of $B_{L}P$, this will be achieved because each peer would send many queries belonging to several other peers. In the case of BTP, this wouldn’t arise because BTP sends queries instead of user information.

#### 4.2.3. Homogeneity

Homogeneity refers to a situation where the points of interests (POIs) or queries of users should be in a different field or major. Thus, Homogeneity is similar to Diversity.

#### 4.2.4. Knowledge about Query Map

If the attacker has knowledge about the query map, it would cause shrinking of the user area; however, it wouldn’t affect the privacy in BTP or $B_{L}P$ because of the congestion of users as the remaining area would increase and so would the level of privacy.

## 5. Analysis of Privacy Factors and Matrices

There are five groups of factors to evaluate and compare privacy approaches [22,24,42,47,48].

### 5.1. Privacy Level

#### 5.1.1. *K*-Anonymity

*K*-Anonymity refers to the number of real queries of a user that attacker has. It is formulated as

(1) K−Anonymity=1/(1+K)

where *K* refers to the number of false queries or dummies, and K≥0.

#### 5.1.2. Entropy

Entropy is the percentage of accurate information as compared to the whole information that the attacker has about the victim. It is a general and accurate metric and other metrics rely on it. The larger the entropy (*E*), the greater the privacy level.

(2) $$E=\sum_{i=0}^{n}(P_{i}*{\left(Log\right)}_{2}\left(P_{i}\right)$$

where $P{}_{i}$ denotes the probability of real query from k queries.

#### 5.1.3. Ubiquity

Ubiquity is related to Entropy; however, it refers to the degree of anonymous data that can’t be attributed to the user without others. A larger value means more privacy level.

(3) $$U=2^{E}$$

#### 5.1.4. Error Estimation

Error Estimation is also related to Entropy; however, it refers to the percentage of error estimation from the attacker about the user according to the information it has.

(4) EE=(E)100%

### 5.2. Need to Trust

We classified the need to trust to five different levels which are: Trust with SP, Trust with TP, Trust with Peer, and Simi-Trust with SP/TP. For trust in all these levels, the user sends part of the data or encrypted (or distorted) data, which is the best choice according to privacy overview, because trusting any party amounts to providing personal information.

### 5.3. Performance

This factor affects the response time, cost of computation, and power consumption. It is also related to many metrics, which are:

- Number of queries that are sent each time to SP.
- Size of transferred data in both the directions, whether the result is for one query, multi-queries, or large part of server data which covers a specific area/cell.
- Using encryption and the type of encryption algorithm, when (a) the asymmetric requests take more time compared to symmetric ones, and (b) symmetric algorithm affect the level of security and the cost of computation affecting time and performance.
- The need for post-processing for the result to find out the final result, as the results after obfuscation queries need some processing to make them suitable for the user, and that involves more cost and time.

### 5.4. Accuracy of Results

Where some approaches and techniques affect the accuracy of relevant results returned by SP, which in turn adversely impacts the evaluation of this technique.

### 5.5. Cache Hit Ratio

It can be added in the approaches that use cache technique to answer future queries and reduce the number of connections with SP. Of course, there is a trade-off between each factor and privacy level factor.

## 6. Superiority of BTP and Blind Peers

As discussed in the previous section BTP/$B_{L}P$ provides the highest level of privacy because, users send their queries to other users instead of sending them to the SP. More and more queries submitted by a user would enhance the privacy pushing the K-Anonymity metric to zero, and Entropy (E) to 1, because all queries with the SP from the same user would have the same probability while in the same time the user ID would be hidden. At the same time U would be maximum (U = 2), and EE of SP would be 100%. The involvement of other attackers wouldn’t arise because of the encryption in both directions.

For Trust Factor, BTP and $B_{L}P$ are also not required to be trusted, which results in achieving the best level of privacy compared to all previous techniques, namely: Dummy, Obfuscation, PIR, and Cache [in the literature review], although some of them have their own methods to avoid trust in other parties.

Because of encryption, the $B_{L}A$ would have lower performance than the one described in [48] or P2PCache [47] approaches, but the computations can be significantly reduced by asymmetric encryption in case when user sends a query with the limited data size whereas in another direction the SP would use symmetric encryption for results such as DES3/AES (Key = 128/192b) with higher level of protection (user changing key each time) and less computation. However, the number of queries sent to the SP in each connection would only be 1, so there would be no need for post-processing of the returned results.

Finally, according to saving the accuracy of result, it can be achieved here. Moreover, the proposed approach can also use the cache techniques. In this case the cache hit-ratio will be maximum because it depended on only real queries of users

## 7. Implementation and Results

Here we provide simulations resulting from comparing figures related to factors, and metrics between $B_{L}A$ and other approaches (Enhances-Cache [42], P2PCache [47], and DCA [48]). We have used MATLAB 2015 in addition to Microsoft Excel 2016 for achieving these simulations. The simulation is based on the following hypotheses, which are same as used in some of the group of seven approaches.

- The study area is divided to 100 × 100 Cells
- There is cache in each access point in each cell.
- The Cache size is 100 K (where POIs of Google-API for the New-York are around 250,000, which required about 250 MB for storage)
- Each query is less than 1 KB in size
- There are 10,000 users distributed randomly in these cells
- There is a list of 100 POIs.
- The network is 3G/4G WI-FI connection in the smart city environment or in specific areas and infrastructure

For the purpose of comparing our approach with the previous ones, we define the following terms which are used as axes.

- K refers to the number of users in the selected area
- Number of queries is the count of queries which are sent to SP in each request
- Average time refers to the required time to search in the cache, small value of which signifies better performance
- Refresh stands for rate of updating the cells in the cache to maintain the threshold of its items
- Response time refers to the average time required to return the result from SP to user, small value of which means better performance
- Entropy refers to the amount of information that the attacker can possibly collect from the queries of a user
- Ubiquity refers to the extent of area of distribution of users in each cell

### 7.1. Average Time vs. Queries Number

**Figure 15:**
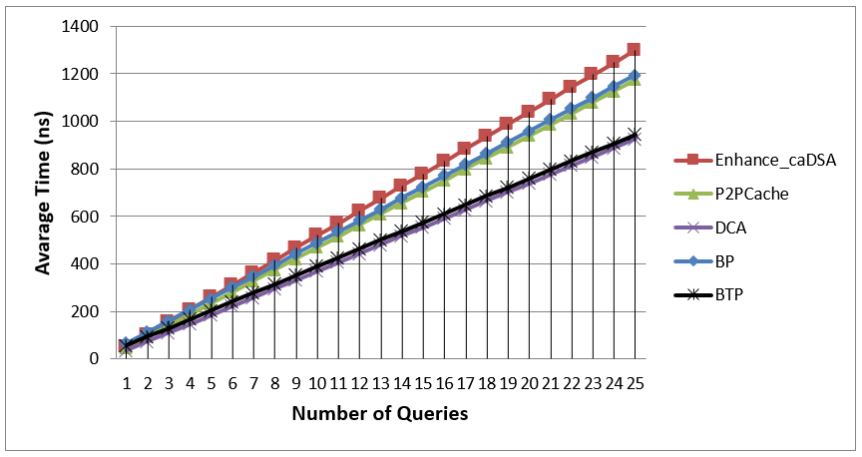
Average Time vs. Number of Queries.

Figure 15 shows that BTP was very close to DCA, and it took the least time, because BTP used encryption which slightly affected the time of each query; however, it still was better than the corresponding time taken by DCA. The same thing happened with $B_{L}P$ and P2PCache but P2PCache was better from $B_{L}P$ because they both used same scenario and $B_{L}P$ used encryption as an additional measure to achieve more privacy.

Enhanced-caDSA was the worst performer because it used an algorithm for generating dummies with higher prediction to be requested in the future, and in the same time it sent many queries to the SP in each connection. Finally, use of encryption in BTP and $B_{L}P$ affected only a little timewise, but enhanced the level of privacy significantly. Moreover, as we explained before, the Blind Approach can be used with the cache technique to enhance the performance if needed.

### 7.2. Response Time

**Figure 16:**
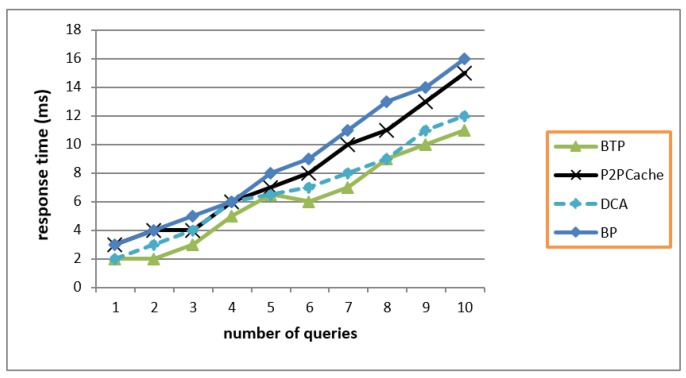
Response Time of user’s queries.

Figure 16 shows that BTP achieved best response time for user’s query, but it came very close to the performance of DCA because the dealing with one party as server in BTP with encryption is surely better than dealing with two caches, and frequently checking whether a query is answered or not. While $B_{L}P$ was the worst performer because it used encryption and in the same time required to search and connect with an available peer impacting on the response time, nevertheless providing the best privacy protection.

### 7.3. Communication Cost

**Figure 17:**
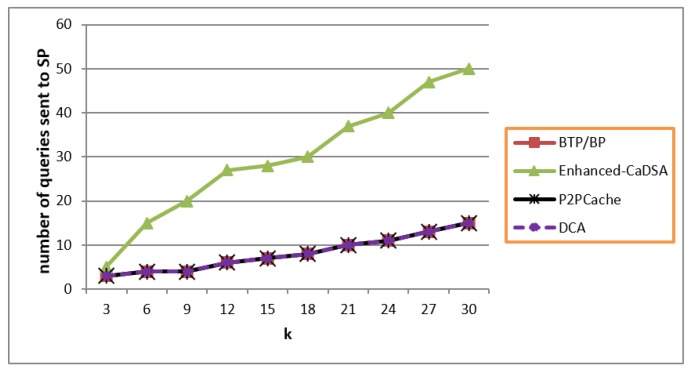
Communication Cost.

Figure 17 shows that Blind Approach (BTP/$B_{L}P$) achieved the lowest cost of number queries alongside DCA and P2PCache. This was because all the techniques sent only one query to SP without any additional dummies or without requesting additional results. Encryption didn’t have any impact on the number of queries but it did (slightly) on the size; which can be interpreted as an improvement towards achieving more security and full privacy.

### 7.4. Privacy Level (Entropy)

**Figure 18:**
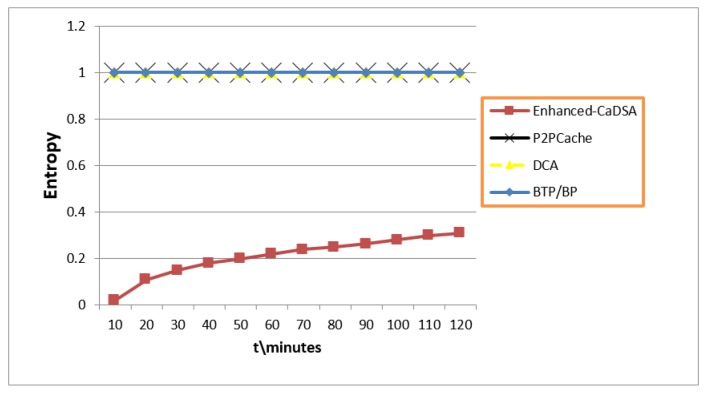
Privacy Factor (Entropy Metric).

As is evident from Figure 18, $B_{L}A$ achieved maximum Entropy (E = 1) because the user didn’t send any personal information. Moreover, $B_{L}A$ supersedes the group of seven approaches because of the Trust Factor.

### 7.5. Privacy Level (Ubiquity)

**Figure 19:**
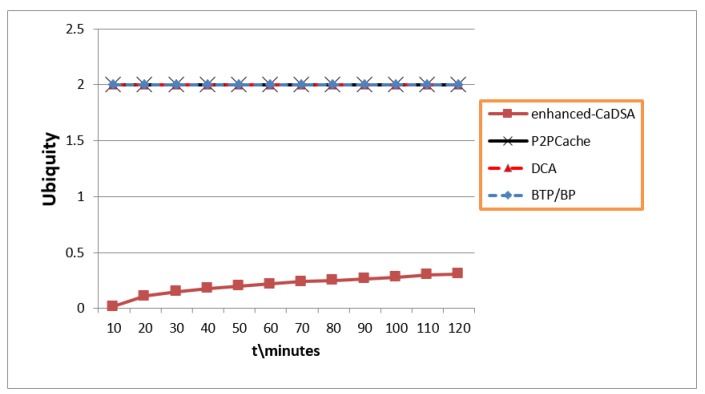
Privacy Factor (Ubiquity Metric).

Figure 19 shows that $B_{L}A$ attains optimum privacy with maximum Ubiquity (U = 2). The reason for this is that each user in $B_{L}A$ sends many queries belonging to different users available everywhere in the cell.

### 7.6. Cache Hit Ratio

**Figure 20:**
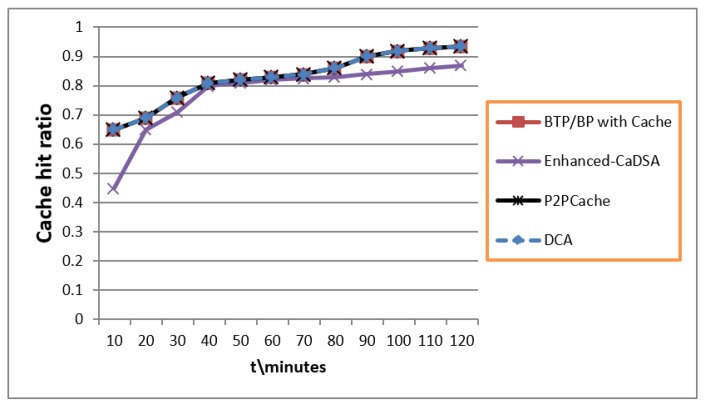
Cache Hit Ratio.

**Figure 21:**
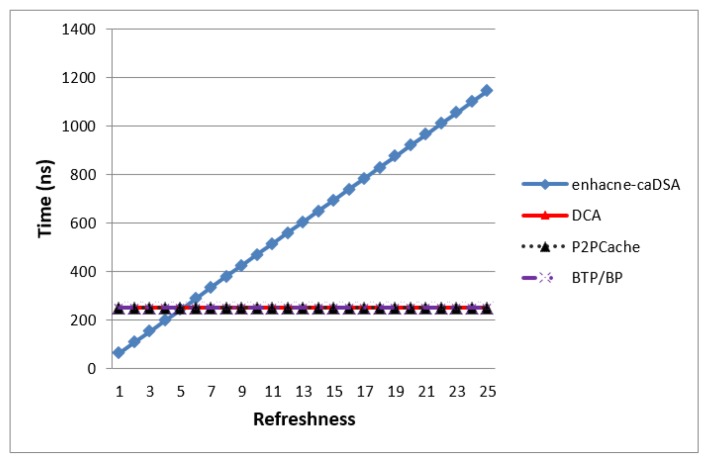
Time of cache management.

As we discussed earlier, $B_{L}A$ can be used with the cache technique for enhancing performance vs. privacy. Figure 20 shows the result in case when $B_{L}A$ with DCA and P2PCache used (same) Algorithm 2 with Bloom Filter and stored only real queries of users in the cache without any dummies. These measures enhanced the hit-ratio and increased the probability of participation of these queries in future. Figure 21 shows that Algorithm 2 of the Blind Approach required less time for managing the cache as compared to Enhanced-CaDSA which uses the freshness method.

## 8. Blind Approach in IoT Applications

Applications of IoT can be found in almost every facet of our life. Let us look at some applications of in some important domains.

### 8.1. Blind Approach and Healthcare Data

Our health is the most important aspect of day to day enjoyment of our lives and is directly linked to the socio-economic and political fabric of the society. With the advent of new technological paradigms, many researchers are suggesting better ways to keep ourselves healthy. In particular, IoT (smart watch, wearable sensors, WSNs, etc.) is providing safe, ubiquitous, and other smarter and ways to better manage health services.

Often health related data is personal and sensitive and requires proper protection. While using the emerging and innovative technologies to manage health, the biggest threat is to preserve privacy and security of the health data. In many applications, health data from devices such as WSNs and RFID tags is continuously streamed to health centers and/or SPs for analysis. Historically, health centers have enjoyed the confidence of protecting health data, but the same cannot be said about SPs.

**Figure 22:**
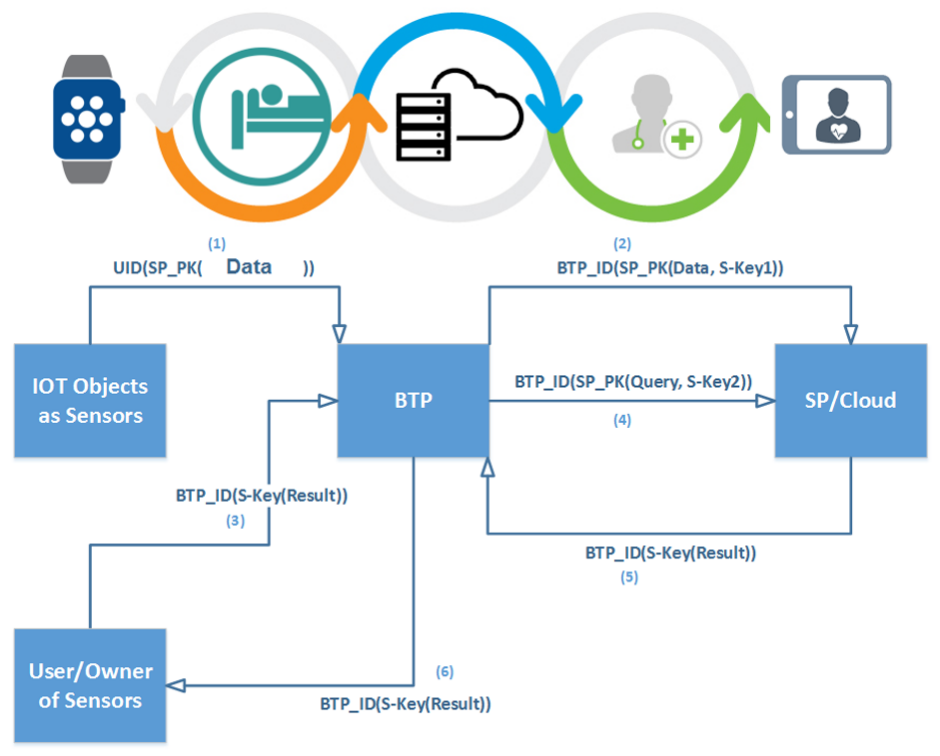
Blind Approach with E-health System.

BTP is the first approach which has proposed efficient and effective solutions in cases such as protection of health data from malicious attacks by SPs themselves. With BTP, users need not disclose the identity to SP or even the health center, and at the same time are not required to reveal to the TP the way it deals with the SP instead of the user. Figure 22 shows working of BTP in this case.

### 8.2. Blind Approach and Smart Transport Systems

**Figure 23:**
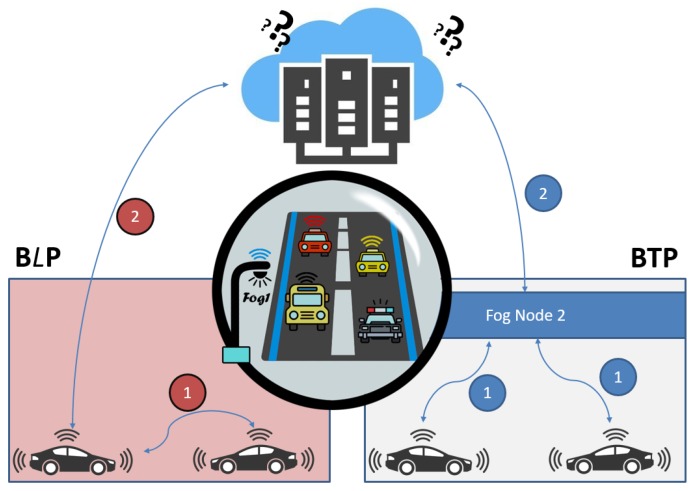
Blind Approach with Transport System.

An important application in the smart city is that of the transportation. Typically, in a smart city, a smart vehicle would send coordinates of its location to SP to seek traffic related information about and coordinates of the points of interest. While obliging with the request, a scrupulous SP with the help of vehicle location at a given time, may detect user’s personal and sensitive information such as home location, habits, movements, workplace, marital status, health condition, religion, etc. Personal information of some individuals may be politically sensitive and linked to their security. Using Bluetooth specifications [49] by generating random IDs (with same location) for a user wouldn’t be effective in this case. It is because the knowledge of location of these IDs with the time of request, would enable SP to detect these IDs, leading to finding the real ID of the user. In case the smart vehicle makes use of Obfuscations or Dummies technique; the accuracy and effectiveness would depend on the number of vehicles in each area. As we know that $B_{L}P$s approach depends on swapping peers before sending to SP. Thus, the number of vehicles wouldn’t affect in any area, as shown in Figure 23. Moreover, SP would collect and store misleading information (places and destinations) about user. It should also be noted that due to swapping, each peer would be blind and unable to do anything beyond reading the query.

### 8.3. Blind Approach and Smart Home

**Figure 24:**
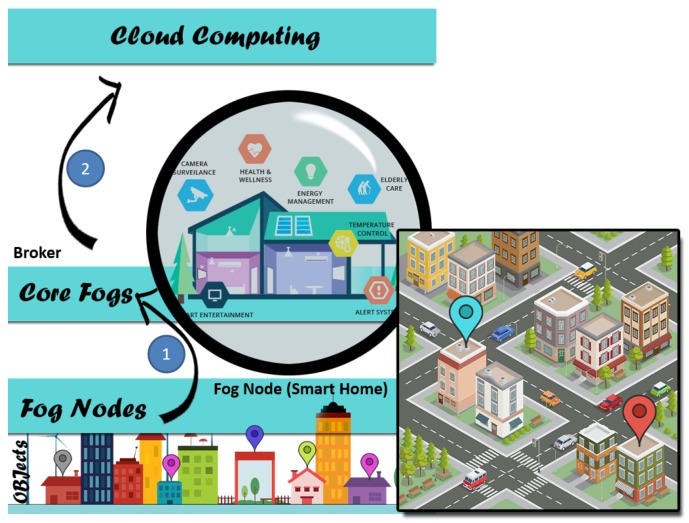
Blind Approach with Smart Home System.

Another application of our approach can be traced in Smart Homes, which use many smart tools such as smart TV, smart light, smart AC, and smart fridge, to name a few. Smart home tools periodically connect to manufactures to enhance the quality of services, which exposes the privacy of users by empowering manufactures to collect a lot of data about users and analyzing it. This data may include home, office, and leisure times of users and their properties. This situation can be avoided by using $B_{L}P$s between different zones of Smart Homes depending upon Fogs [50]; and using BTP, depending on the core fog, as shown in Figure 24.

### 8.4. Blind Approach and Underwater Wireless Sensor Network

**Figure 25:**
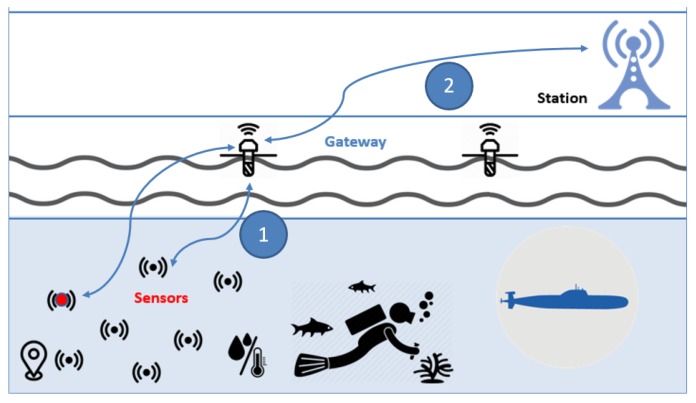
Blind Approach with Underwater System.

Another application of Blind Approach [51] used by military or a monitoring organization searching for oil or fossil fuels is shown in Figure 25. Here $B_{L}A$ can be used to prevent eavesdropping, and malicious node accessing data from other sources. $B_{L}A$ can also be used to thwart attacks in this environment such as the attack that monitors the most active node to get information about the location of the main station. In other words, the swapping method in many situations can prevent the attacker to achieve its goal.

### 8.5. Comparison of Performance of Blind Approach with the Group of Seven Approaches

**Figure 26:**
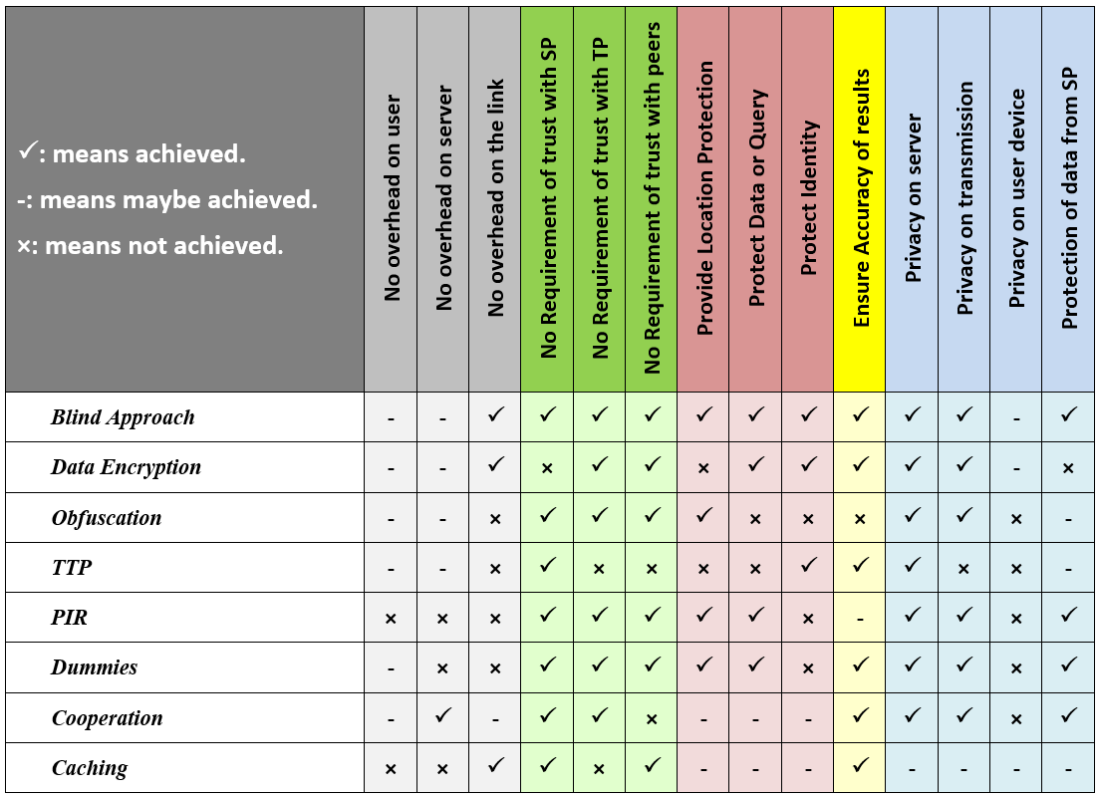
Performance of Blind Approach vs. Group of Seven Approaches.

Figure 26 provides a summary of performance of the Blind Approach compared with the group of seven approaches. As is evident, Blind Approach has significant advantages over the group of seven approaches.

Here it should be noted that we are not claiming that $B_{L}A$ can solve all the problems of privacy in all IoT applications; nevertheless, as demonstrated in this article, it provides protection for some applications where the group of seven approaches fail to do so. Indeed, so far, there isn’t an approach which would work in all situations. To streamline what could be done in different situations, we are working on a framework which would contain many techniques, including $B_{L}A$, which would automatically select suitable technique(s) for each application or service. As a drawback of our approach, it should be noted that there is a possibility of breaking into BTP in scenarios such as that in the case of health. This will happen if TP and SP collate to disclose the identity and other information of the user. Although such would be a rare occasion, nevertheless it would be probable. We intend to return to this aspect and propose a resolution in the future.

## 9. Conclusions

In this paper, we have introduced $B_{L}A$ as a superior approach for preserving privacy in IoT applications, which has three scenarios to provide effective and efficient solutions in different cases. Our approach amicably resolves the trust issue as users can protect their privacy without having to trust the third party, which has been a challenging open problem in all methods relying on third party trust. The $B_{L}A$ also introduces a new method for sending dummies to the SP without an overhead on the user, server, or network. In addition, $B_{L}A$ provides accuracy in returning the result back to the user.

The simulation and results of comparison demonstrate superiority of $B_{L}A$ over the group of seven approaches, with a slight increase in the average (response) time. This can be viewed as a trade-off between the average (response) time and privacy and security of data. As observed in the simulation results, BTP/$B_{L}P$ achieved the highest level of privacy without relying on the third-party trust or compromising on performance, cost, or accuracy. We have seen that our approach supports many applications of IoT, and also provides flexibility to use the idea of BTP/$B_{L}P$ with the group of seven approaches.

In the future, we intend to provide enhanced protocols for addressing the privacy issue with the M-Health application, and also to focus on critical applications of underwater wireless sensors. We also endeavor to show how we can detect any attempt from BTP to get information about any user. We shall also try to provide a way to reduce the effect of encryption on performance by confining its applications to the most sensitive part (instead of the whole query), and deal with the drawbacks of $B_{L}A$.
